# Inhibitory Effects of Standardized *Leonurus japonicus* Extract and Its Bioactive Leonurine on TNF-α-Induced Muscle Atrophy in L6 Myotubes

**DOI:** 10.4014/jmb.2005.05023

**Published:** 2020-06-19

**Authors:** Jiyeon Lee, Changhee Kim, Hyerin Lee,, Jae-Kwan Hwang

**Affiliations:** 1 Department of Biomaterials Science and Engineering, Yonsei University, Seoul 03722, Republic of Korea; 2 R&D Center, FND Net, Seoul 05706, Republic of Korea; 3 Department of Biotechnology, College of Life Science and Biotechnology, Yonsei University, Seoul 0722, Republic of Korea; 4 Graduate Program in Bioindustrial Engineering, Yonsei University, Seoul 03722, Republic of Korea

**Keywords:** Leonurine, *Leonurus japonicus*, muscle atrophy, skeletal muscle

## Abstract

Muscle atrophy, characterized by a reduced number and size of myofibers, occurs due to immobilization, aging, and several chronic diseases. *Leonurus japonicus*, belonging to the Labiatae family, is widely used as a traditional medicine in Korea, China, and Japan. Previous studies have reported that *L. japonicus* has various physiological activities, such as anti-bacteria, anti-cancer, and liver protection. Leonurine, which is a major bioactive in *L. japonicas*, is known to possess biological effects including anti-inflammation, anti-fibrosis, anti-angiogenesis, and anti-diabetes. However, the preventive effects of *L. japonicas* and leonurine on muscle have not been reported. The current study aimed to determine the inhibitory effects of standardized *L. japonicus* extract (LJE) and leonurine on muscle atrophy by clarifying their underlying molecular mechanisms in tumor necrosis factor-alpha (TNF-α)-stimulated L6 myotubes. LJE and leonurine stimulated the phosphatidylinositol 3-kinase/Akt pathway that was reduced by TNF-α treatment. LJE and leonurine not only increased the mammalian target of rapamycin pathway for protein anabolism but also decreased the mRNA expression of E3 ubiquitin ligases by blocking the translocation of Forkhead box O, which is closely linked with proteolysis. Additionally, LJE and leonurine alleviated inflammatory responses by downregulating TNF-α and interleukin-6 mRNA expression and reducing the protein expression of nuclear factor-kappa B, a major transcriptional factor of proinflammatory cytokines. Collectively, LJE and leonurine have potential as therapeutic candidates for inhibiting the development of skeletal muscle atrophy by activating the PI3K/Akt pathway and reducing inflammatory responses.

## Introduction

Skeletal muscle, known as a protein reservoir, is part of an organ system that comprises 40% of body weight and plays roles in maintaining body posture, performing physical activities, and metabolizing carbohydrates, proteins, and fats [1]. Muscle atrophy, defined as decreased muscle fiber area, density, or mass, occurs under various conditions including aging, disease, and physical inactivity [2]. Loss of skeletal muscle is a serious concern since it not only leads to muscle malfunction and reduced therapeutic responses, but also severely complicates metabolic diseases such as obesity and diabetes [3,4]. Ultimately, muscle atrophy devastates the quality of life of affected individuals, consequently leading to increased mortality and morbidity [4].

Muscle mass depends on protein turnover which is determined by an elaborate and complicated balance between protein synthesis and degradation [5]. Once this balance is disturbed by detrimental and negative factors, such as proinflammatory cytokines, protein formation is decreased while proteolysis is increased, which ultimately reduces net protein contents and instigates muscle wasting [5, 6]. In consideration of protein degradation, the ubiquitin-proteasome system is a main system that determines the rate of proteolysis. E3 ubiquitin ligases, muscle atrophy F-box (MAFbx; also known as atrogin-1) and muscle RING finger 1 (MuRF1), are potent and major inducers of proteolysis in the ubiquitin-proteasome system [3, 7]. Following their polyubiquitination by E3 ubiquitin ligases, target proteins, such as myosin heavy chains, are bound to the proteasome and hydrolyzed into small peptides [7]. In contrast to MuRF1 and atrogin-1, the mammalian target of rapamycin (mTOR) is a major anabolic biomarker for protein. Activated mTOR phosphorylates the 70-kDa ribosomal protein S6 kinase (p70S6K) and eukaryotic initiation factor 4E-binding protein 1 (4E-BP1) to stimulate translation [8]. Thus, targeting and regulating these protein turnover-related pathways have been suggested to decelerate the loss of protein content in muscle.

The phosphatidylinositol 3-kinase (PI3K)/Akt pathway is a major signaling cascade responsible for regulating protein translation and proteolysis [8]. As a downstream factor of insulin-like growth factor-1 (IGF-1), which is a protein responsible for muscle growth, PI3K activates Akt through phosphorylation [5, 9]. Activated Akt not only phosphorylates mTOR, subsequently stimulating p70S6K and 4EBP-1 for protein anabolism, but also plays an important role in stimulating the phosphorylation of cytoplasmic Forkhead box O3 (FoxO3), which inhibits its nuclear translocation [10]. FoxO3 protein works as a transcriptional factor to regulate E3 ubiquitin ligase genes and autophagy-related genes [6]. Once FoxO3 translocation is blocked by Akt phosphorylation, FoxO3 cannot regulate its target genes, thereby suppressing the subsequent stimulation of protein degradation-related systems [7, 10]. Thus, the PI3K/Akt pathway, which reverts the abnormal protein turnover-related pathways to normal states, can be a therapeutic signaling target for developing agents against muscle atrophy.

**Fig. 1:**
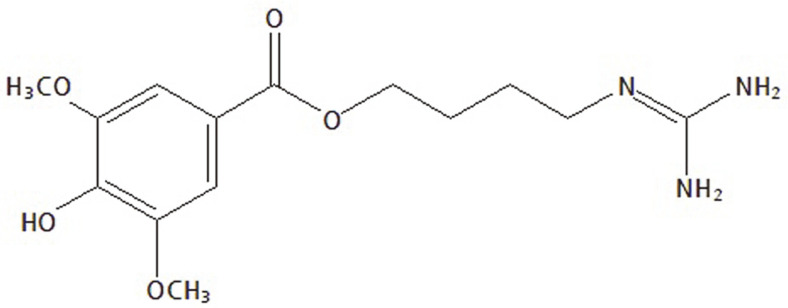
Chemical structure of leonurine.

*Leonurus japonicus* Houtt. (common name: Chinese motherwort), is widely used as a traditional herbal medicine for several symptoms and diseases, such as menstrual irregularities, edema, and ulcers [11]. Previous studies have demonstrated the physiological, pharmacological, and biological activities of *L. japonicus*, such as, anti-cancer, anti-bacteria, and vasorelaxation [11, 12]. Leonurine (4-guanidio-n-butyl-syringate) (Fig. 1), a type of alkaloid, is a major active compound in *L. japonicus* and has been reported to exhibit various biological activities, including anti-inflammation, anti-fibrosis, anti-angiogenesis and anti-diabetes [13]. *L. japonicus* and leonurine have commonly shown beneficial effects on several organs, including the liver, brain, and smooth muscle [12, 13]. However, their protective effects on skeletal muscle have not been elucidated. In this study, we investigated whether standardized *L. japonicus* extract (LJE) and leonurine attenuated muscle atrophy by activating the PI3K/Akt pathway in tumor necrosis factor alpha (TNF-α)-stimulated L6 myotubes.

## Materials and Methods

### Preparation and Standardization of LJE

Dried *L. japonicus* leaves were ground and then extracted in water at 70°C for 4 h. After filtration, water was removed with a rotary vacuum evaporator (Heidolph Instruments GmbH & Co., KG., Germany). The yield of obtained *L. japonicus* extract was 13.67%. *L. japonicus* extract was standardized with leonurine using high-performance liquid chromatography (YL9100 HPLC system; Younglin Instruments Co., Ltd., Korea) equipped with a Sunfire C18 column (5 μm i.d., 150 × 4.6 mm; Waters, USA). Gradient mobile phases comprising 1%phosphoric acid (A) and methanol (B) were as follows: 95% A for 0-10 min, 80% A for 10-20 min, 65% A for 20-30 min. Wavelength and flow rate were set at 277 nm and 1.0 ml/min, respectively. After plotting a standard curve for leonurine (Chemfaces, China) with various concentrations versus peak area, the chromatographic peak of leonurine in *L. japonicus* extract was identified by confirming its retention time. Based on the standard curve, the concentration of leonurine in LJE was calculated. LJE contained 0.98% (w/w) leonurine as a bioactive compound.

### Cell Culture

L6 rat myoblasts were purchased from the American Type Culture Collection (USA) and cultured at 37°C and 5% CO_2_ in Dulbecco’s modified Eagle’s medium (DMEM; Hyclone, USA) containing 10% fetal bovine serum (FBS; Hyclone). Myoblast differentiation into myotubes was performed according to a previous study [6]. Briefly, to induce differentiation, 10% FBS (Hyclone) in DMEM (Hyclone) was exchanged with 2% horse serum (Hyclone) in DMEM. This medium was replaced every two days for 6 days. On day 6, the cells were cotreated with both 50 ng/ml TNF-α (PeproTech, USA) and LJE or leonurine for 12 h.

### Cell Viability

Cytotoxicity was evaluated using 3-(4,5-dimethylthiazol-2-yl)-2,5-diphenyltetrazolium bromide reagent (MTT; Sigma-Aldrich, USA). After treatment with LJE or leonurine for 24 h, the cells were incubated with 300 μl of 0.5 mg/ml MTT solution at 37°C for 3 h. After aspiration of MTT solution, the formed MTT-formazan product was dissolved in dimethyl sulfoxide, and the absorbance was measured at 540 nm with the VersaMax™ tunable microplate reader (Molecular Devices, Inc., USA).

### Measurement of Myotube Diameter

The morphologies of myotubes were observed using a CK40 inverted microscope (Olympus, Japan) equipped with a T500 camera (magnification, ×200; eXcope, Korea). The random areas of each group were captured and the diameters were measured using the Image J software (National Institutes of Health, USA).

### Western Blot Assay

Cell lysates were prepared using the NP-40 buffer solution (Elpis-Biotech, Korea) containing 0.2% proteinase inhibitor cocktail (Sigma-Aldrich). Protein concentration in each sample was quantified with the Bradford reagent (Bio-Rad Laboratories, USA). After boiling at 95°C for 5 min, equal protein quantities were separated on sodium dodecyl sulfate-polyacrylamide gel electrophoresis, and then the separated protein was transferred to a nitrocellulose membrane (Whatman GmBH, Germany). Primary antibodies were reacted with the protein on the nitrocellulose membrane at 4°C for 20 h. Nuclear factor kappa B (NF-κB) primary antibody was purchased from Santa Cruz Biotechnology (USA). Primary antibodies for p-PI3K, PI3K, p-Akt, Akt, p-FoxO3, FoxO3, p-mTOR, mTOR, p-p70S6K, p-4E-BP1, 4E-BP1, and α-tubulin were purchased from Cell Signaling Technology (USA). After washing three times for 10 min with Tris-buffered Saline Tween 20 (TBST), the membrane was incubated with horseradish peroxidase-conjugated anti-rabbit or anti-mouse secondary antibody (Bethyl Laboratories, Inc., USA). After washing three times for 10 min with TBST, the membrane was developed using an enhanced chemiluminescence detection reagent (GE Healthcare, USA), and the protein bands were identified using the G:BOX EF imaging system (Syngene, UK). Protein intensities were quantified using the Image J software (National Institutes of Health).

### Reverse Transcription-Polymerase Chain Reaction (RT-PCR)

Total RNA isolation from the cells and RT-PCR analysis were performed using TRIzol reagent (Takara, Japan) and the Gene Amp PCR System 2700 (Applied Biosystems, USA), respectively. Isolated total RNA was quantified using the Nanodrop 1000 spectrophotometer (Thermo Scientific, USA). The cDNA was synthesized with Reverse Transcriptase Premix (Elpis-Biotech) and total RNA at 42°C for 55 min and 70°C for 15 min. PCR was carried out with SafeDry Taq PCR premix (CellSafe, Korea) and specific primers (Bioneer, Korea) of which sequences are shown in Table 1. Thermal profile for target gene amplification was as follows: 95°C for 10 min for enzyme activation and 35 cycles of denaturing at 95°C for 30 s, annealing at 58-60°C for 30 s, and elongating at 72°C for 45 s. The amplified cDNA was stained with 5× Loading STAR dye (DyneBio, Korea) and separated by electrophoresis with 1.5% agarose gel. The cDNA band was identified using the G:BOX EF imaging system (Syngene). The band densities were quantified using the Image J software (National Institutes of Health).

### Statistical Analysis

The experiments were performed independently at least three times. All data are expressed as mean ± standard deviation and were statistically evaluated with one-way analysis of variance, followed by Duncan’s multiple range test using the SPSS 25.0 software (IBM Corp., USA). Significant difference between groups was considered at *p* < 0.05.

## Results

### Effects of LJE and Leonurine on Cytotoxicity

**Fig. 2:**
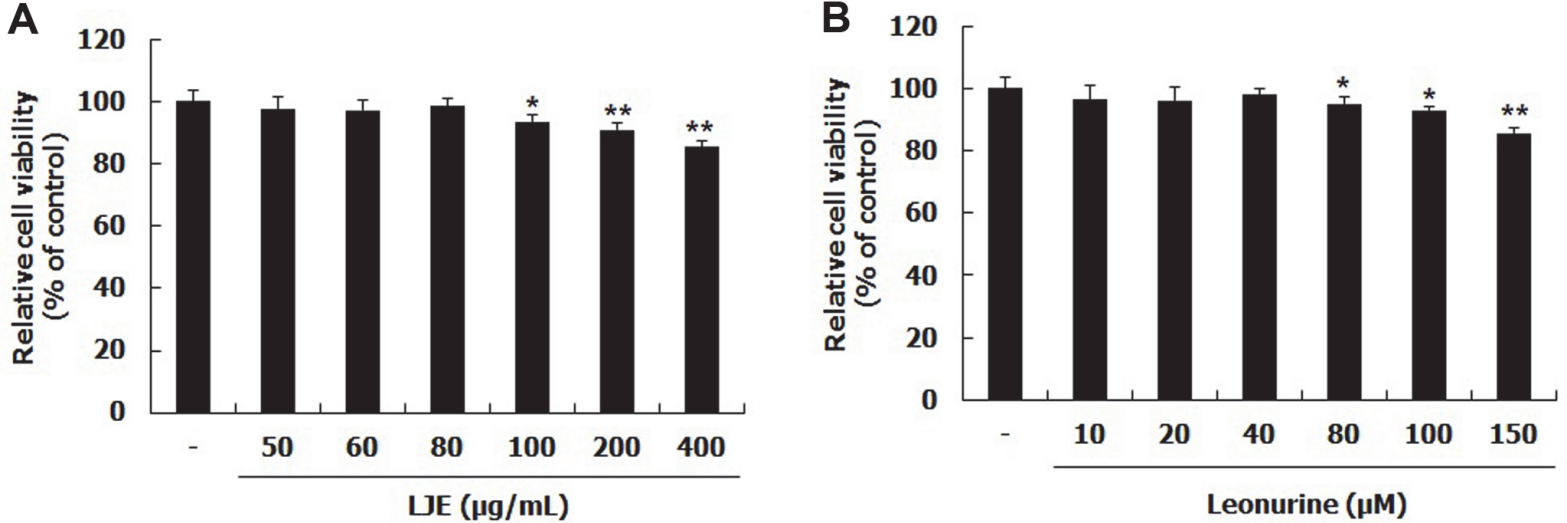
Effects of LJE and leonurine on cell viability of L6 myotubes. L6 myotubes were treated with LJE (from 50 to 400 μg/ml) and leonurine (from 10 to 150 μM). Relative cell viability was tested using MTT regents (**A, B**). Group differences were assessed by Duncan’s multiple range test, *p < 0.05, and **p < 0.01 (control group vs. LJE- and leonurine-treated groups).

First, the cytotoxic effects of LJE and leonurine on L6 myotubes were evaluated with MTT assay. After differentiation, cells were treated with LJE or leonurine at various concentrations. LJE treatment at more than 100 μg/ml showed significantly reduced cell viability (Fig. 2A); leonurine treatment less than 40 μM did not significantly reduce cell viability (Fig. 2B). Thus, further experiments were conducted with 40 and 80 μg/ml LJE and 20 and 40 μM leonurine.

### Effects of LJE and Leonurine on Myotube Diameter

**Fig. 3:**
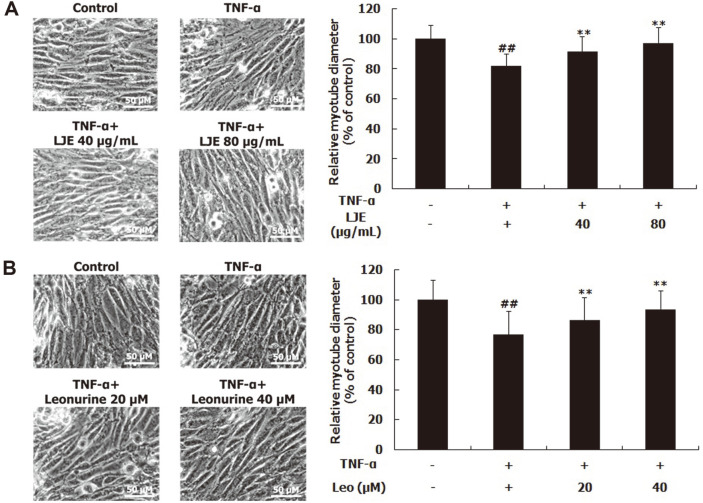
Effects of LJE and leonurine on the diameters of L6 myotubes. L6 myotubes were treated with TNF-α (50 ng/ml), LJE (40 and 80 μg/ml), and leonurine (20 and 40 μM). Relative myotube diameters were measured (**A, B**). Group differences were assessed by Duncan’s multiple range test, ##*p* < 0.01 (negative control group vs. TNF-α-treated group), **p < 0.01 (TNF-α-treated group vs. LJE- and leonurine-treated groups).

To investigate whether LJE and leonurine inhibited TNF-α-induced muscle atrophy, the diameters of myotubes were measured. TNF-α treatment significantly reduced the diameter of myotubes, compared to the untreated control (Figs. 3A and 3B). However, LJE at 40 and 80 μg/ml increased the reduced diameter of myotube by 11.60% and 18.78%, respectively (Fig. 3A). The treatment of leonurine at 20 and 40 μM also raised it by 13.05% and 21.95%, respectively (Fig. 3B).

### Effects of LJE and Leonurine on Muscle Protein Synthesis-Related Biomarkers

**Fig. 4:**
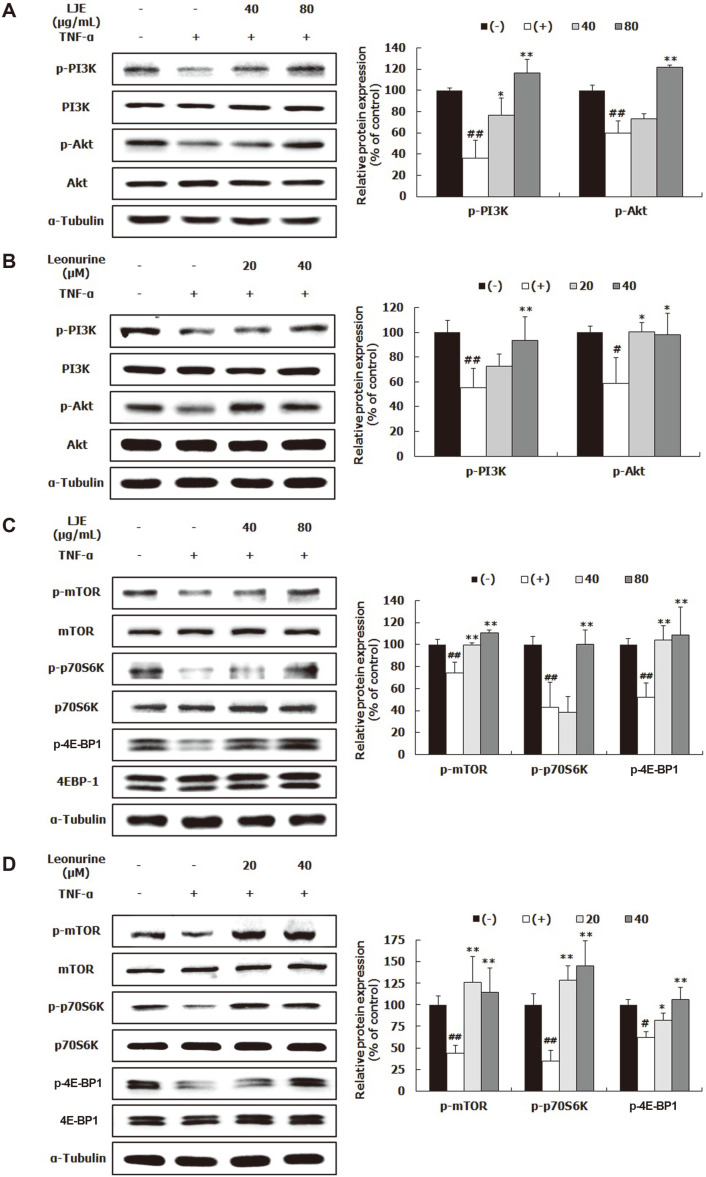
Effects of LJE and leonurine on muscle protein synthesis in L6 myotubes. L6 myotubes were treated with TNF-α (50 ng/ml), LJE (40 and 80 μg/ml), and leonurine (20 and 40 μM). Relative protein levels of p-PI3K, PI3K, p-Akt, and Akt (**A, B**) were measured using western blot, with α-tubulin as the housekeeping gene. Relative protein levels of p-mTOR, mTOR, p-p70S6K, p70S6K, p-4E-BP1, and 4E-BP1 (**C, D**) were evaluated using western blot, with α-tubulin as the housekeeping gene. Group differences were assessed by Duncan’s multiple range test, #*p* < 0.05 and ##*p* < 0.01 (negative control group vs. TNF-α-treated group), *p < 0.05, and **p < 0.01 (TNF-α-treated group vs. LJE- and leonurine-treated groups).

To determine whether LJE and leonurine inhibited muscle atrophy, their effects on muscle protein turnover-related biomarkers were evaluated. Western blot analysis was performed to examine whether LJE and leonurine enhanced the PI3K/Akt pathway, an essential regulator of protein synthesis and protein degradation [7]. In the TNF-α-treated control, p-PI3K and p-Akt were remarkably reduced, compared to the untreated control (Figs. 4A and 4B). LJE dose-dependently increased p-PI3K expression; however, p-Akt protein expression was markedly upregulated only at an LJE concentration of 80 μg/ml but not 40 μg/ml (Fig. 4A). Similarly, leonurine also raised the PI3K protein expression in a concentration-dependent manner; however, a significant difference was only found with 40 μM leonurine (Fig. 4B). Leonurine treatment significantly elevated p-Akt protein expression. Additionally, mTOR is responsible for protein anabolism in muscle and is a downstream factor of the PI3K/Akt pathway [7,14]. TNF-α significantly reduced both p-mTOR and its downstream factors, p-p70S6K and p-4E-BP1 (Figs. 4C and 4D). Moreover, p-mTOR and p-4E-BP1 protein expression was significantly upregulated in response to LJE treatment, compared to that of TNF-α-treated control (Fig. 4C). Regarding p-p70S6K protein expression, a remarkable increase was only observed with 40 μg/ml LJE. The protein expression levels of p-mTOR, p-p70S6K, and p-4E-BP1 in leonurine-treated groups were remarkably higher than those in the TNF-α-treated control (Fig. 4D).

### Effects of LJE and Leonurine on Proteolysis-Related Biomarkers

**Fig. 5:**
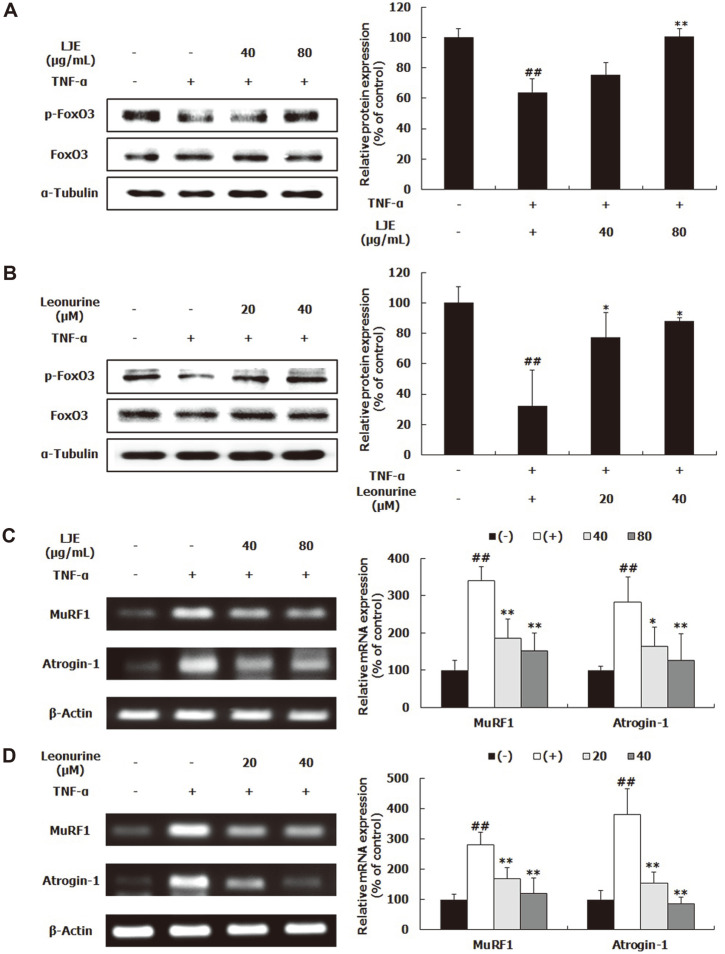
Effects of LJE and leonurine on muscle protein degradation in L6 myotubes. L6 myotubes were treated with TNF-α (50 ng/ml), LJE (40 and 80 μg/ml), and leonurine (20 and 40 μM). Relative protein levels of p-FoxO3a and FoxO3a (**A, B**) were measured using western blot, with α-tubulin as the housekeeping gene. Relative mRNA levels of atrogin-1 and MuRF-1 (**C, D**) were evaluated using RT-PCR, with β-actin as the housekeeping gene. Group differences were assessed by Duncan’s multiple range test, ##*p* < 0.01 (negative control group vs. TNF-α-treated group), *p < 0.05 and **p < 0.01 (TNF-α-treated group vs. LJE- and leonurine-treated groups).

FoxO3, which is modulated by the PI3K/Akt pathway, regulates the transcription of target genes [8]. If FoxO3 in the cytoplasm is phosphorylated by activated Akt, it cannot pass into the nucleus, resulting in reduced protein degradation [6]. The expression of p-FoxO3 in the TNF-α-treated control group was significantly decreased, compared to the control group (Figs. 5A and 5B), indicating that the FoxO3 migrates more into the nucleus when the cells were exposed to TNF-α. After treatment with LJE and leonurine, p-FoxO3 protein expression, which was decreased by TNF-α, was significantly increased (Figs. 5A and 5B). Next, the mRNA expression of MuRF1 and atrogin-1 was verified with RT-PCR. As a result, the mRNA expression levels of MuRF1 and atrogin-1 were significantly upregulated by TNF-α treatment, whereas LJE and leonurine treatments drastically reduced these levels in a dose-dependent manner (Figs. 5C and 5D).

### Effects of LJE and Leonurine on Inflammatory Response

**Fig. 6:**
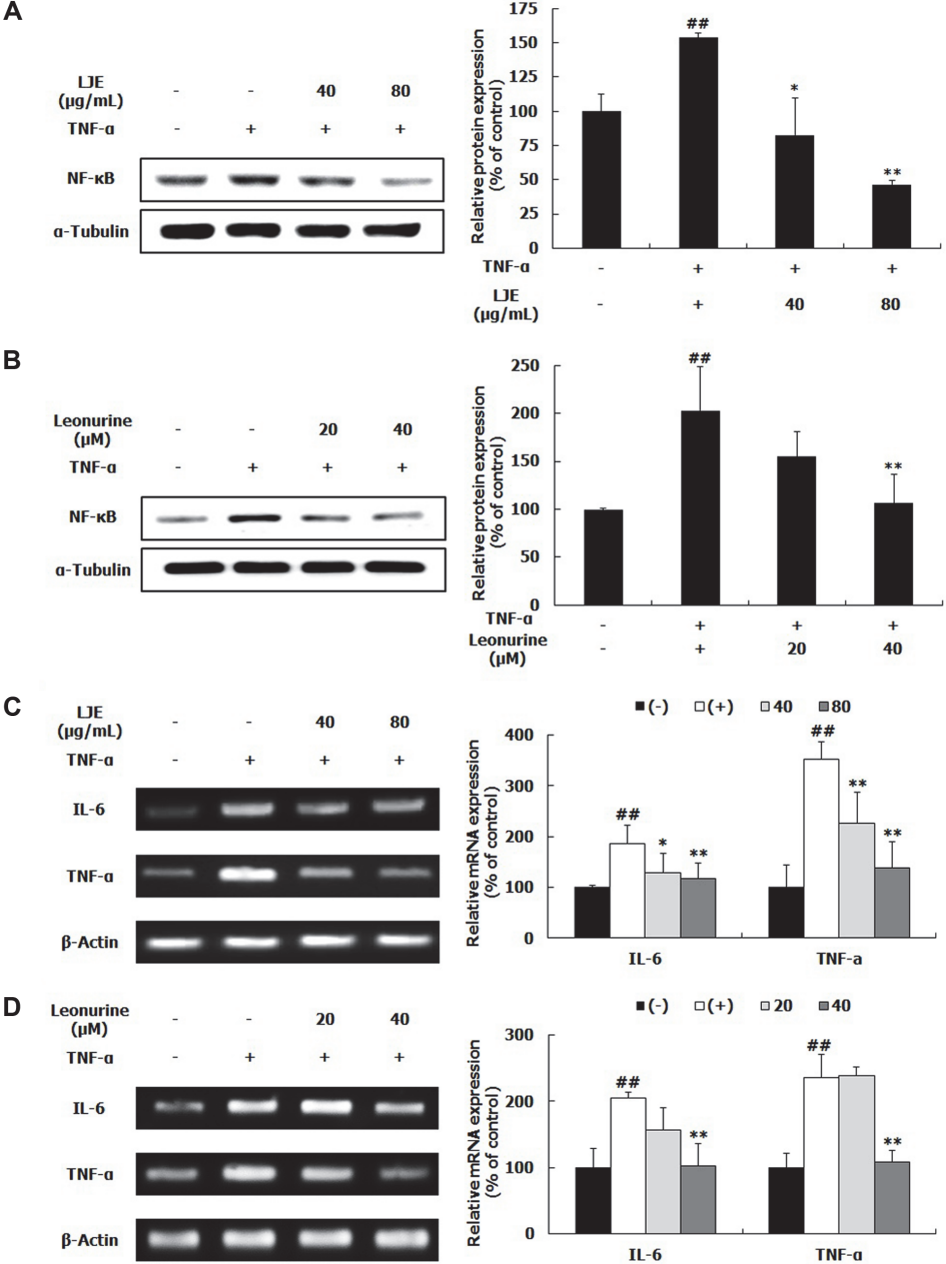
Effects of LJE and leonurine on inflammatory response in L6 myotubes. L6 myotubes were treated with TNF-α (50 ng/ml), LJE (40 and 80 μg/ml), and leonurine (20 and 40 μM). Relative protein levels of NF-κB (**A, B**) were measured using western blot, with α-tubulin as the housekeeping gene. Relative mRNA levels of TNF-α and IL-6 (**C, D**) were evaluated using RT-PCR, with β-actin as the housekeeping gene. Group differences were assessed Duncan’s multiple range test, ##*p* < 0.01 (negative control group vs. TNF-α-treated group), *p < 0.05 and **p < 0.01 (TNF-α-treated group vs. LJE- and leonurine-treated groups).

NF-κB is a major inflammation-related transcription factor that regulates inflammatory cytokines [15]. In this study, NF-κB protein expression and the mRNA expression levels of interleukin (IL)-6 and TNF-α were confirmed by western blot and RT-PCR, respectively. TNF-α treatment dramatically increased NF-κB protein expression (Figs. 6A and 6B). However, LJE and leonurine treatments greatly reduced TNF-α-induced NF-κB expression. In particular, when treated with 80 μg/ml LJE, the average expression of NF-κB was lower than that of the control group (Fig. 6A). Similarly, when cells were treated with TNF-α, the mRNA expression levels of IL-6 and TNF-α were significantly upregulated, compared to the control group; meanwhile, the upregulated IL-6 and TNF-α mRNA expression was markedly reduced by LJE and leonurine (Figs. 6C and 6D).

## Discussion

Muscle atrophy is characterized by reduced muscle mass and muscle proteins. Several extrinsic and intrinsic factors, including aging, disuse, and chronic diseases, such as AIDS, obesity, and cancer stimulate the process of muscle wasting through inflammatory responses [5, 6]. Physiologically, TNF-α and IL-6, which represent main proinflammatory cytokines, are well-known catabolic factors involved in instigating muscle atrophy [3, 15]. Thus, several studies have employed TNF-α-treated cells as an in vitro model for research on muscle atrophy. TNF-α treatment reduced the diameters of L6 rat and C2C12 mouse myotubes [6, 8]. TNF-α treatment also disturbed cell proliferation and fusion in satellite cells isolated from horse muscle which are cells contributing to muscle hypertrophy [16]. To ascertain whether LJE and leonurine had inhibitory activities on muscle atrophy, TNF-α-treated L6 myotubes were used as a muscle atrophy model in this study. TNF-α treatment decreased the diameter of L6 myotubes, compared to the non-treated control group; however, LJE and leonurine reversed the reduced diameter of L6 myotubes (Figs. 3A and 3B). These results indicate that LJE and leonurine have an ability to inhibit muscle atrophy.

The PI3K/Akt pathway plays an important role in protein turnover. When the pathway was activated by IGF-1, muscle growth subsequently increased [17]; however, once p-PI3K and p-Akt levels have been decreased by TNF-α, muscle fiber diameter becomes significantly reduced [6, 8]. In this study, TNF-α treatment decreased p-PI3K and p-Akt protein expression (Figs. 4A and 4B), indicating that TNF-α disturbs the PI3K/Akt pathway in L6 myotubes and stimulates muscle wasting. These results are consistent with previous studies [6, 8]. Here, LJE and leonurine reversed the TNF-α-induced reduction in p-PI3K and p-Akt (Figs. 4A and 4B). In agreement with these results, leonurine activated the PI3K/Akt signaling pathway in H_2_O_2_-stimulated H9c2 cardiac myocytes [18]. Akt regulates protein formation and degradation by activating the mTOR/p70SK6/4E-BP1 pathway and blocking the translocation of FoxO3 [14, 19]. In this study, LJE and leonurine increased the protein expression of p-mTOR, p-p70S6K, and p-4E-BP1 (Figs. 4C and 4D). Consistently, leonurine activated the mTOR/extracellular signal-regulated kinase (ERK) pathway for angiogenesis and tissue regeneration [20]. In addition, LJE and leonurine treatments increased p-FoxO3 protein expression and reduced MuRF1 and atrogin-1 mRNA expression (Fig. 5), indicating that LJE and leonurine prevent the transcription of E3 ubiquitin ligases by blocking FoxO3 translocation. Thus, these results suggest that LJE and leonurine possess the potential to inhibit muscle atrophy by targeting the PI3K/Akt pathway and subsequently improving protein turnover-related pathways. Additionally, the stimulatory effect of LJE on the PI3K/Akt pathway is partially attributed to leonurine.

In addition to the PI3K/Akt pathway, other possible pathways involve molecules that could serve as targets for the development of potential agents against muscle atrophy [21]. One negative physiological change occurring in atrophic muscle is mitochondrial dysfunction [1]. Leonurine protected mitochondria function and prohibited the mitochondrial-dependent apoptotic pathway in rat brains [22], suggesting that leonurine improves mitochondrial function and mitochondrial-dependent apoptosis in atrophic skeletal muscle. Further studies are required to reveal other possible molecular targets that are involved in the inhibitory effects of LJE and leonurine on muscle atrophy.

In this study, TNF-α treatment increased NF-κB, IL-6, and TNF-α expression, indicating that TNF-α-induced NF-κB levels upregulate the mRNA expression of TNF-α and IL-6 (Fig. 6). Inflammatory cytokines, such as TNF-α and IL-6, are well-known inducers of muscle atrophy as well as inflammatory response [3, 4]. TNF-α, which binds to membrane receptors of muscle cells, stimulates the NF-κB signaling pathway [15]. The activated NF-κB signaling pathway not only raises the mRNA expression of E3 ubiquitin ligases genes, but also upregulates the expression of cytokines [15, 23]. Phytochemicals or plant extracts with anti-inflammatory activities have been examined to reveal their potential to inhibit muscle atrophy development and delay the rate or degree of muscle wasting [3, 21]. In this study, LJE and leonurine significantly inhibited the expression of NF-κB, TNF-α, and IL-6 (Fig. 6). Consistently, LJE attenuated acute inflammation by inhibiting the production of inflammatory cytokines, IL-1β, TNF-α, and prostaglandin E2 and NF-κB-involved inducible nitric oxide synthase in lipopolysaccharide (LPS)-stimulated RAW264.7 cells [24]. Leonurine also reduced the LPS-induced TNF-α, IL-6, and IL-8 levels, together with the inactivation of NF-κB signaling pathway [25]. Thus, the anti-inflammatory properites of LJE and leonurine are partially involved in their anti-atrophic effects on muscle.

Previous studies have presented the beneficial effects of alkaloid compounds on muscle function and muscle physiology. Tetrahydropalmatine, an alkaloid compound isolated from *Corydalis turtschaninovii*, stimulated myoblast differentiation through Akt and p38 activation [26]. Tomatidine increased the myotube diameter in C2C12 cells as well as grip strength and muscle mass in mice [27]. In this study, leonurine improved protein turnover-related pathways in L6 myotubes. Thus, alkaloid compounds have a potential to block the development of muscle atrophy, which offers insights into the potential use of alkaloid compounds to develop synthetic chemical compounds against muscle wasting. In addition to alkaloids, several other compounds have been identified in *L. japonicus* [11, 12]. Quercetin inhibited muscle atrophy in obese mice by reducing inflammatory response and E3 ubiquitin ligase expression [2]. Apigenin has conferred muscle hypertrophic and anti-atrophic effects in animal models [28, 29]. Ferulic acid also stimulated muscle growth in zebrafish through increasing p-p70S6K and p-4E-BP1 [30]. Thus, it is conceivable that the anti-atrophic effect of LJE on muscle results from the combination of phytochemicals in extract including leonurine. However, it is unclear whether this mixture exerts synergistic effect in extract; thus, further study is necessary.

In this study, LJE and its active compound, leonurine, improved protein turnover-related pathways in TNF-α-stimulated L6 myotubes. LJE and leonurine not only activated the mTOR/p70SK6/4E-BP1 pathway for protein synthesis, but also suppressed FoxO3 translocation and the expression of E3 ubiquitin ligases. In LJE and leonurine-mediated improvement of protein-turnover pathways, the PI3K/Akt pathway was mainly involved target proteins. Additionally, LJE and leonurine attenuated inflammatory responses by downregulating NF-κB, TNF-α, and IL-6. Taken together, LJE and leonurine are promising agents for the treatment of skeletal muscle atrophy. In addition, the current study provides a potential direction for further investigation regarding the roles of LJE and leonurine as therapeutic agents against muscle atrophy in animal models and clinical trials.

## Figures and Tables

**Table 1:** Primer sequences used in RT-PCR analysis.

Gene	Direction	Sequence (5’-3’)
MuRF1	Forward	CCG GAC GGA AAT GCT ATG GA
	Reverse	AGC CTG GAA GAT GTC GTT GG
Atrogin-1	Forward	ATG TCT GGA GGT CGT TTC CG
	Reverse	CGT CTT CGT GTT CCT TGC AC
TNF-α	Forward	GGC TTT CGG AAC TCA CTG GA
	Reverse	CCC GTA GGG CGA TTA CAG TC
IL-6	Forward	TGT TCT CTA CCG AAG AAC TGG CAA TA
	Reverse	GAA ACC ATC TGG CTA GGT AAG AGA A
β-Actin	Forward	TGA CAG GAT GCA GAA GGA GAT
	Reverse	TAA AAC GCA GCT CAG TAA CAG
